# Predictors of care coordination versus social visitation with long-term care facility residents

**DOI:** 10.1093/geroni/igaf125

**Published:** 2026-03-04

**Authors:** Caroline Collins-Pisano, Rachel Weiskittle

**Affiliations:** Department of Psychology, University of Colorado Colorado Springs, Colorado Springs, Colorado, United States; Department of Psychology, University of Colorado Colorado Springs, Colorado Springs, Colorado, United States

**Keywords:** Residential care, Loneliness, Social support, Family caregiving

## Abstract

**Background and Objectives:**

Most studies to date have assessed visitation between family and residents via the overall frequency of in-person visits. This approach fails to account for 2 different types of involvement included in visitation: care coordination and social visitation. This study aimed to identify facilitators and barriers of care coordination distinct from social visitation with a specific focus on long-term care (LTC) site characteristics and resident functionality.

**Research Design and Methods:**

One hundred and seventy-five adult participants in the United States with a close friend or relative residing in a LTC facility completed an online survey regarding their care coordination and social visitation in LTC facilities, LTC facility characteristics, and residents’ functionality.

**Results:**

Shorter travel time and visitors’ experiences communicating with the resident (e.g., comfort, frustration) emerged as the strongest predictors of both care coordination involvement and social visitation. Both greater comfort communicating with the resident and greater frustration predicted greater care coordination involvement, whereas only comfort communicating with the resident predicted social visitation. Resident length of stay, type of LTC facility, and resident communication factors (e.g., verbal abilities, recognition of visitors) were not significant predictors of care coordination involvement nor social visitation.

**Discussion and Implications:**

Logistical barriers and visitors’ emotional experiences when communicating with residents may have a stronger influence on visitor engagement compared to resident’s functional abilities and LTC facility characteristics. Interventions targeting the identified facilitators of visitation may help to facilitate and support family involvement with residents of LTC facilities.

Innovation and Translational Significance Research often treats social visitation and care coordination as a single construct, limiting our understanding of the contextual and interpersonal influences of family visitation with long-term care (LTC) residents. This study differentiates the two and identifies predictors of each form of involvement. Findings inform strategies to foster meaningful connection and continuity of care for LTC residents, supporting the development of organizational and policy-level interventions that target barriers to care coordination and social visitation (e.g., improving access to asynchronous and hybrid visitation tools and educating family members on communicating with those with dementia), ultimately promoting more meaningful involvement in LTC settings.

## Background and objectives

According to the most recent cycle of the CDC’s National Post-acute and Long-term Care Study, over 800,000 older adults were living in residential care communities in 2022, a number expected to rise significantly in the coming decades (Melekin et al., 2024; National Center for Health Statistics [NCHS], 2023). By 2050, one-third of Americans aged 65 years or older are predicted to relocate to a senior living community, resulting in more than a 20% increase in the LTC population (Chaulagain et al., 2021). Dementia is highly prevalent in these settings, with approximately 44% of residents diagnosed with Alzheimer’s disease or other forms of dementia, making it the second most common chronic condition in LTC residents after hypertension (58%; NCHS, 2023).

LTC residents face a heightened risk of social isolation due to physical, cognitive, and environmental barriers that limit engagement in previously enjoyed social activities (Boamah et al., 2021). As a result, social well-being is especially critical in institutional settings, where it plays a key role in promoting healthy aging and quality of life (National Academies of Sciences, Engineering, and Medicine [NASEM], 2020). Visitation by family and friends supports residents’ well-being by reducing loneliness, improving mental health, and slowing cognitive and functional decline (Abbott et al., 2018; Cohen-Mansfield & Parpura-Gill, 2007). Ongoing contact with loved ones also reinforces self-identity and provides essential emotional and practical support (Falk et al., 2013; Fitzpatrick & Tzouvara, 2019; Roberts & Ishler, 2018).

National survey data indicate that while most LTC residents receive some form of visitation, considerable variation exists across facilities, and little is known about the quality or frequency of these interactions (NCHS, 2023). Residents are more likely to receive visits when they live close to family or when their length of stay is shorter (Roberts & Ishler, 2018; Weimer et al., 2022). However, communication barriers often lead to reduced or discontinued visitation, especially in cases of advanced dementia. Visitors may feel emotional distress when residents can no longer engage in conversation or recognize them (Boamah et al., 2021). Caregivers of older adults with dementia report more emotional difficulty than caregivers of those without dementia due to these communication barriers (Freedman et al., 2022).

Much of the existing research conceptualizes LTC involvement as a single dimension, typically focused on visitation frequency. This approach overlooks the diverse ways in which loved ones engage with residents. Emerging work has identified two distinct domains: social visitation and care coordination involvement (Weimer et al., 2022). Social visitation encompasses emotionally supportive interactions such as shared meals, conversations, and participating in recreational or religious activities, whether in person or virtually. Care coordination involvement pertains to more instrumental roles, such as monitoring care quality, advocating for the resident, and communicating with staff (Klostermann & Funk, 2022; Powell et al., 2018; Ris et al., 2019; Roberts et al., 2020). These functions are conceptually and practically distinct, but few studies have examined them separately or considered how different factors may shape involvement in each.

To address these gaps, this study examined both social visitation and care coordination involvement among family members and friends of LTC residents. Using a national survey sample, we investigated how facility characteristics (e.g., type of facility, geographic distance, length of stay) and resident-level communication factors (e.g., verbal communication ability, recognition of visitors, cognitive status) predicted engagement across these two domains. By identifying distinct predictors and barriers to each form of LTC involvement, this study aims to inform efforts that support meaningful connection and continuity of care for older adults in LTC settings.

## Research design and methods

The results of these analyses are taken from an Institutional Review Board (IRB) approved Qualtrics survey study on overall barriers and facilitators of friend and family social visitation and care coordination involvement with LTC residents (#2025-087-ONLINE).

### Participants and procedure

Two-hundred-and-fifty-five participants were recruited via ResearchMatch, a national health volunteer registry, to complete an online survey. Individuals were eligible to participate if they were aged 18 years or older and reported having a close friend, relative, or loved one currently living in LTC. Upon survey completion, participants were entered into a drawing to receive one of five $20 gift cards.

### Measures

#### Care coordination involvement

The Care Coordination Involvement subscale (CCIS) was created by the authors for the purpose of this study from the Family Involvement Questionnaire-Long-Term Care (FIQ-LTC; Fast et al., 2019). The FIQ-LTC is a 40-item self-report measure developed to assess the ways in which the respondent interacts with the resident in LTC settings. Internal consistency was found to be highly reliable with a Cronbach’s α of 0.97 (Fast et al., 2019). For the present study, the scale’s original questions were modified to read “loved one” rather than “family member” in order to account for a wider range of participants’ relationships to the LTC resident. Twenty-six items were selected for the CCIS to measure care coordination visitation, such as attending treatment team meetings and communicating resident health information to LTC staff. Sample items include “I talk to facility staff to ensure my loved one has access to stimulating activities” and “I assist my loved one in managing their finances.” Subscale scores range from 26 to 104, with higher scores indicating higher coordination of care. Internal consistency for items was high in this sample, demonstrating a Cronbach’s α of .97.

#### Social visitation

Social visitation was measured via the Social Involvement Subscale (SIS), created by the authors for the purpose of this study from the FIQ-LTC (Fast et al., 2019). The remaining 14 items of the FIQ-LTC were selected for the SIS to measure social visitation with the LTC resident. Sample items include “I come and have meals with my loved one” and “I attend activities offered by my loved one’s facility.” Subscale scores range from 14 to 56, with higher scores indicating more frequent social visitation. The SIS demonstrated relatively high internal consistency in the study’s sample with a Cronbach’s α of .87.

#### LTC facility characteristics

The LTC Facility Characteristics Questionnaire was developed for the purpose of this study to assess characteristics of the residents’ LTC facility. The three-item multiple-choice questionnaire assessed the resident’s current living situation, including the type of facility they resided in (i.e., independent or assisted living facility, nursing home or skilled nursing facility, or memory care unit), the resident’s length of stay in the facility (0–5 years, or more than 5 years), and the travel time from the participants’ home to the LTC facility (0–8 hr, or more than 8 hr).

#### Communication factors

The Resident Communication Factors Questionnaire was developed to assess characteristics of the residents’ communication abilities and visitor’s experiences when communicating with the residents. Within the five-item multiple-choice questionnaire, participants were asked to report on their loved one’s ability to communicate, alertness, and recognition of the participant. Sample items included, “Is your family member, friend, or loved one currently able to communicate verbally,” “Does your family member, friend, or loved one currently recognize you when you are together,” and “How often is your family member, friend, or loved one currently alert and aware of your interactions together.” Responses ranged from 1 (*never*) to 5 (*always*). The three questions were summed to create a composite score representative of the resident’s communication abilities. Possible composite scores ranged from three to 15, with higher scores representing greater communication abilities.

The Visitor Communication Experience Questionnaire is a brief, 2-item questionnaire created for the purpose of the study to assess participants’ comfort and level of frustration interacting with the resident, respectively. Items included “How comfortable are you communicating and interacting with your family member, friend, or loved one?” and “How frustrated do you become when interacting with your family member, friend, or loved one?” Responses to both items ranged from 1 (*not at all*) to 7 (*extremely*), with higher scores representing greater comfort or feelings of frustration when communicating with the LTC resident.

All measures used in the survey study, including items and scoring details, are provided in the online supplementary material.

### Data analyses

Data analyses were performed using SPSS version 29.0.2.0 (IBM Corp). Descriptive statistics and correlational analyses were computed to characterize the sample. A multiple regression was run to evaluate the relationship between LTC facility characteristics, resident communication factors, visitor communication experiences, and the outcome variable of care coordination involvement. A second multiple regression was run to evaluate the relationship between LTC facility characteristics, resident communication factors, visitor communication experiences, and the outcome variable of social visitation.

## Results

### Data preparation

A total of 255 participants were recruited for the study. Participants were excluded if they declined the consent (*n *= 4), did not know someone well residing in a LTC facility at the time of the survey (*n *= 13), completed less than 80% of the survey (*n *= 25), failed one or more attention check (*n *= 31), completed the survey in less than 8 min (*n *= 3), or demonstrated repeated IP addresses (*n *= 4). A final total of 175 participants were included in the study (Supplementary Figure 1, see online supplementary material).

Prior to analysis, study variables were examined for outliers, defined as a *z*-score greater than 3 or less than −3, and missing data. The continuous variable of *minutes traveled to a LTC Facility* was capped at “8+ hours.” These responses were removed from analyses, including this variable, for accuracy (*n *= 24, 14%). Three variables within the Resident Communication Factors Questionnaire offered “I don’t know” responses (i.e., variables of recognition [*n *= 4], alertness and awareness [*n *= 2], and type of facility [*n *= 3]); participants who endorsed this response were excluded from analyses involving these variables.

### Descriptive data

Participants’ ages ranged from 19 to 81 (*M *= 49.58, *SD *= 17.86). The sample consisted of 129 females (74%), 43 males (24%), and four participants who identified as an “other gender” (2%). The majority of participants were White (*n *= 142, 81%). Approximately 5% of participants identified as Black, 3% as Hispanic, 3% as multiracial, 1% as American Indian, 1% as Middle Eastern, and 1% as Native Hawaiian. Two participants reported that their race was not listed (1%), and one participant preferred to not report their race (1%). Further demographic information and relational characteristics of the study participants, including frequencies, means, and standard deviations, are provided in Table 1. Further characteristics of the study’s measures, including means, standard deviations, internal consistencies, and skewness values, are provided in Tables 2–4.

**Table 1.** Demographic and relational characteristics of study participants.

Demographic variable	*N*	%	Mean	*SD*
**Gender**				
** Male**	4	24		
** Female**	129	74		
** Other gender**	4	2		
**Race/Ethnicity**				
** American Indian**	2	1		
** Asian**	5	3		
** Black**	9	5		
** Hispanic**	6	2		
** Middle Eastern**	1	1		
** Native Hawaiian**	1	1		
** White**	142	81		
** Multiracial**	2	1		
** Race not listed**	1	1		
** Prefer not to say**	6	3		
**Relationship status**				
** Single**	47	27		
** Married/long-term relationship**	112	64		
** Widowed/separated/divorced**	16	9		
**Employment status**				
** Full-time/part-time/student**	108	62		
** Unemployed**	18	10		
** Retired**	49	28		
**Household income**				
** Less than $25,000**	18	10		
** $25,000–$49,999**	26	15		
** $50,000–$74,999**	37	21		
** $75,000–$99,999**	20	11		
** $100,000–$149,999**	30	17		
** $150,000 or more**	27	15		
** Prefer not to say**	17	10		
**Age**			49.58	17.86
**Closeness to resident**			3.66	1.00
**Relation to resident**				
** Grandparent**	55	31		
** Mother**	30	17		
** Father**	13	7		
** Uncle or aunt**	10	6		
** Spouse**	5	3		
** Siblings**	8	5		
** Friend**	25	14		
** Other extended family**	29	17		

**Table 2.** Characteristics of outcome subscales.

Characteristics	*M*	*SD*	Cronbach’s α	Skewness
**Coordination of care involvement subscale^a^**	62.55	22.82	0.97	0.09
**Social involvement subscale^a^**	36.79	8.60	0.87	−0.22

a Subscales were developed from the Family Involvement Questionnaire-Long-Term Care.

**Table 3.** LTC facility characteristics.

Characteristics	*M*	*SD*	*N*	%
**Years living in facility**	2.63	2.91		
**Type of facility**				
** Independent/assisted living**			71	41
** Nursing home/skilled nursing**			66	38
** Memory care unit**			35	20
** Other**			3	2
**Minutes traveled to LTC facility^a^**	98.51	130.2		

*Note.* LTC = long-term care.

a This continuous variable was capped at “8+ hours”; these responses were removed from analyses for accuracy (*n *= 24).

**Table 4.** Resident and visitor communication factors.

Factors	*M*	*SD*
**Resident communication factors^a^**	12.53	2.80
**Participant comfort communicating**	3.77	1.00
**Participant frustration communicating**	2.07	0.96

a Composite score created from the summation of resident verbal communication, recognition, and alertness variables.

### Bivariate correlational analysis

Bivariate correlational analyses for all continuous predictive and outcome variables are provided in Table 5. Of interest, the outcome variables of care coordination involvement and social visitation were significantly positively correlated, *r*(173) = .73, *p* < .01, demonstrating a large effect size and significant shared variance and significant non-shared variance. Resident communication factors were significantly positively correlated with social visitation, *r*(173) = .25, *p* < .01 but were not associated with care coordination involvement, *r*(173) = −.01, *p* > .05.

**Table 5.** Correlations table.

Variable	1	2	3	4	5	6
**1. Coordination of care involvement**						
**2. Social visitation**	.73**					
**3. Years living in facility**	−.10	−.03				
**4. Minutes traveled to LTC facility**	−.20*	−.20**	.07			
**5. Resident communication factors**	−.01	.25**	−.04	−.07		
**6. Participant comfort communicating**	.20**	.41**	.07	−.05	.41**	
**7. Participant frustration communicating**	.14	−.05	−.15	−.04	−.07	−.32**

*Note*. LTC = long-term care.

* *p* < .05.

** *p* < .01.

### Multivariate regression analysis

#### Care coordination involvement

A linear regression examined predictors of care coordination involvement. The model was statistically significant, explaining 13% of the variance, with a medium effect size *F*(7, 159) = 3.26, *p* < .01 (Supplementary Table 1, see online supplementary material). In the model, shorter travel time (β = −0.20, *p* < .01), greater frustration communicating with the resident (β = 0.22, *p* < .01), and greater comfort communicating with the resident (β = 0.26, *p* < .01) uniquely predicted greater care coordination involvement. Length of resident stay in the facility (β = −0.09), facility type (independent or assisted living facility vs. memory care unit: β = 0.07, nursing home or skilled nursing facility vs. memory care unit: β = 0.13), and resident communication factors (β = −0.14) were not significant in the model.

#### Social visitation

Second, a linear regression examined predictors of social visitation. The model was statistically significant, explaining 20% of the variance, with a medium effect size *F*(7, 159) = 5.69, *p* < .001 (Supplementary Table 2, see online supplementary material). In the model, shorter travel time (β = −0.18, *p* < .05) and greater comfort communicating with the resident (β = 0.35, *p* < .001) uniquely predicted greater social visitation. Length of resident stay (β = −0.03), facility type (independent or assisted living facility vs memory care unit: β = 0.14, nursing home or skilled nursing facility vs memory care unit: β = 0.19), frustration when communicating with the resident (β = 0.08), and resident communication factors (β = 0.06) were not significant in the model.

## Discussion and implications

This study examined how facility- and resident-level characteristics predict two distinct but related forms of family involvement in LTC: care coordination involvement and social visitation. Care coordination involvement pertains to instrumental tasks that support LTC residents’ well-being, such as monitoring care quality and communicating with staff, whereas social visitation refers to emotionally supportive tasks such as sharing meals, co-participating in activities, and conversing with the resident (Weimer et al., 2022). By incorporating both contextual and interpersonal predictors of these two forms of involvement, our study offers a more nuanced understanding of how families visit and engage with residents. Several key patterns emerged regarding LTC facility characteristics, resident communication factors, and visitor communication experiences, with travel time and visitor communication experiences serving as the most influential predictors.

### Care coordination involvement

Travel time was a significant barrier to care coordination involvement. Participants with greater travel time between their home and the LTC facility had lower care coordination involvement than those with less travel time. This finding aligns with prior research associating shorter commutes with more frequent LTC visits, regardless of transportation mode (Miller, 2019, 2020; Weimer et al., 2022). The travel time of this study’s sample was approximately an hour and a half on average (*M *=  98.51 min), with high variability (*SD *= 130.29). These figures are commensurate with larger-scale studies. This pattern suggests that logistical challenges, such as a lengthy commute, have a pivotal impact on family members’ involvement in LTC settings. Asynchronous communication tools, proactive outreach, and hybrid visitation models are among some of the possible strategies staff can engage in to help attenuate this impact.

Visitor communication experiences also played a central role in predicting care coordination involvement. Participants who reported greater comfort communicating with the resident also reported higher levels of care coordination involvement. This result is consistent with prior literature emphasizing that LTC visits are more likely to continue when they are perceived as positive or rewarding (Cohen-Mansfield & Parpura-Gill, 2007). Unexpectedly, greater frustration communicating with the resident was also associated with greater care coordination involvement. Although frustration stemming from communication challenges related to dementia has been linked to negative outcomes such as caregiver burden and depression (e.g., Petrovsky et al., 2020; Volkmer et al., 2024), our finding suggests that withdrawal from care coordination involvement is not among these outcomes. Instead, visitors appear to maintain their caregiving efforts despite the associated interpersonal difficulties. Providing families with communication coaching, space to process their emotional experiences, and validation of the difficulty of maintaining connection in the face of cognitive decline may help reduce distress among visitors and promote sustained involvement.

### Social visitation

Travel time was a significant barrier to social visitation, such that longer commutes were predictive of significantly less social visitation. Thus, families who live farther away face challenges not only in serving instrumental care coordination roles but also in maintaining relational connections with the resident. This pattern supports previous evidence that longer commutes negatively impact overall visitation frequency and involvement in LTC (e.g., Weimer et al., 2022), but considering social visitation separate from care coordination offers unique insights into this trend. Despite having more opportunities to visit, shorter-distance visitors did not appear to place greater emphasis on social visitation than care coordination, or vice versa. Instead, shorter travel time facilitated greater involvement across both domains. Future research could explore this dynamic further by prospectively evaluating family members’ activities during LTC visits along with their relative travel times.

### Broader patterns across both forms of LTC involvement

Comfort communicating with the resident was the strongest predictor of both care coordination and social visitation, reinforcing the importance of emotionally meaningful visitor–resident interactions in maintaining LTC involvement (Cohen-Mansfield & Parpura-Gill, 2007). In contrast, greater frustration communicating with the resident predicted greater care coordination but did not predict social visitation. This finding suggests that family members may pivot negative emotions related to communication challenges into tangibly productive care tasks, without significantly disengaging socially from the resident.

The composite score of resident communication factors (comprised of verbal communication ability, alertness, and recognition of visitors) was significantly positively correlated with social visitation but was not associated with care coordination involvement. The predictive value of residential communication factors on social visitation diminished once visitor comfort communicating was considered. Although prior research has found that family members experience greater emotional and communicative strain as residents’ cognitive capacities decline, which can reduce the frequency or quality of visits (Boamah et al., 2021; Freedman et al., 2022), our results indicate that subjective emotional experiences communicating with the resident influence visitor engagement more than the resident’s functional abilities.

Finally, LTC facility characteristics, such as length of stay and type of facility, were neither associated with nor predictive of either form of LTC involvement. Similar to the above findings, family members’ interpretation and perception of their visit experiences appear to be stronger predictors of involvement compared to LTC setting characteristics. Interventions targeting visitor emotional experiences and logistical barriers, such as geographic distance, may help to facilitate care coordination and social visitation.

## Limitations and future directions

This study has several limitations. First, it used a cross-sectional survey design, which precludes any causal inferences between identified barriers and visitation behaviors. Without temporal data, it remains unclear how distinct forms of interaction influence each type of involvement over time. Future research should employ longitudinal designs to better capture how these variables relate over time. Longitudinal studies could explore, for instance, how changes in residents’ functioning affect family members’ care coordination involvement.

Second, the sample has limited generalizability due to a lack of demographic diversity in the sample. The majority of participants identified as White (81%). Cultural factors such as one’s ethnic/racial background may impact LTC involvement. For example, Thomeer et al. (2018) found that Hispanic and Black older adults were less likely to move to an LTC facility compared to White older adults, attributable in part to cultural beliefs about aging and familial caregiving responsibilities. Li and Cai (2014) found that racial and ethnic minority residents experienced significantly lower levels of social engagement in nursing home facilities compared to White residents. Furthermore, participants were recruited from a national online database of research volunteers (ResearchMatch), potentially resulting in a skewed sample of individuals with higher-than-­average computer literacy. Although our results demonstrate a normal distribution of socioeconomic factors in this sample, future studies should evaluate our findings using multi-faceted research strategies outside of virtual recruitment sources.

The findings of this study offer several directions for future research. Consistent with prior research, travel time emerged as a key predictor of LTC involvement across domains. Further investigation of its impact on visitation is of growing importance. Rising costs and labor shortages are diminishing the availability of local LTC facilities in the United States, contributing to longer LTC commutes for families across diverse regions (Sharma et al., 2024). Existing research in this area has been hindered by inconsistent measurement (e.g., distance vs travel time), limiting comparability across studies (Mseke et al., 2024).

Further research on visitors’ subjective emotional experiences when interacting with LTC residents is warranted. Caregiver support resources frequently emphasize strategies for reducing frustration through effective communication techniques (e.g., Family Caregiver Alliance, n.d.), but other communication-related emotions remain less explored in the literature, such as comfort, anxiety, and sorrow. As studies continue to reveal the salience of family members’ emotions in the context of LTC involvement, closer examination of these constructs will improve our understanding of their influence on family members’ decision-making.

Findings from this study can help inform the development of interventions aimed at optimizing family involvement in LTC and reducing barriers to visitation. Along these lines, experimental studies are needed to test causal mechanisms linking visitation patterns with LTC resident loneliness and social isolation. Research should also continue to explore the ways in which care coordination involvement and social visitation influence not only family involvement but also residents’ mental health outcomes, as these domains may have distinct impacts on health indices.

## Supplementary Material

igaf125_Supplementary_Data

## Data Availability

Our data are not available to other researchers at this time as the authors have not completed their original work with the data set. The research was not pre-registered.
