# Supplementary material for: Predictors of care coordination versus social visitation with long-term care facility residents
Source: Innov Aging. 2026 Mar 4;10(3):igaf125. doi: 10.1093/geroni/igaf125 (PMC12965327; doi:10.1093/geroni/igaf125)
Supplement: igaf125_Supplementary_Data [file igaf125_supplementary_data.zip › innage suppl Collins-Pisano and Weiskittle.docx]

***Innovation in Aging* Supplementary Material: Collins-Pisano & Weiskittle. Predictors of Care Coordination versus Social Visitation with Long-Term Care Facility Residents.**

**Supplementary Figure 1.** Participant Exclusion Flow Chart


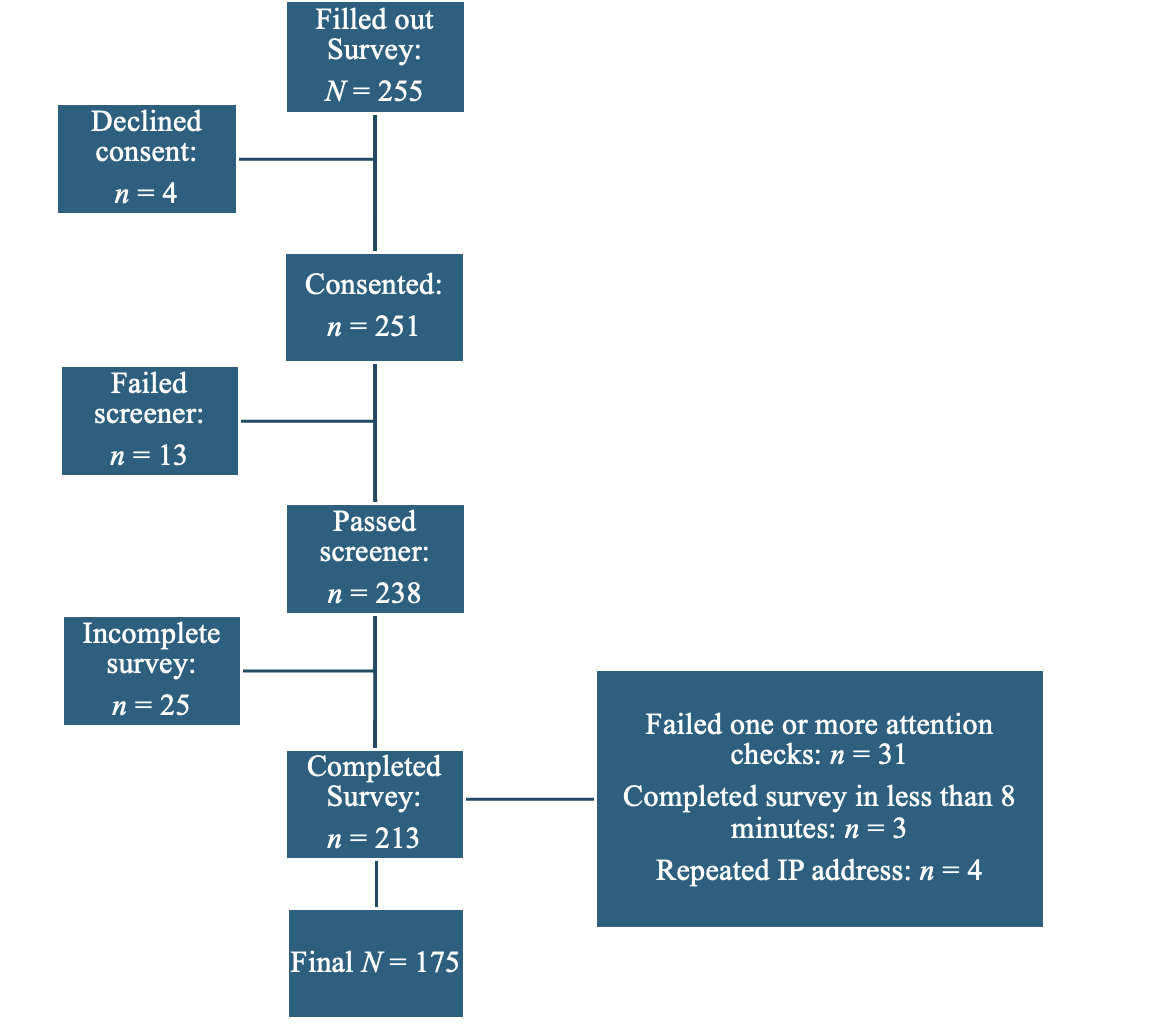


**Regression Tables**

**Supplementary Table 1.** *Multiple Regression Model: Care Coordination Involvement*

| Predictor | *B* | *SE* | ß |
| --- | --- | --- | --- |
| Years Living in Facility | -0.65 | 0.58 | -.09 |
| Minutes Traveled to LTC Facility | -0.03 | 0.01 | -.20** |
| Type of Facility: Independent/Assisted Living | 3.21 | 5.08 | .07 |
| Nursing Home/Skilled Nursing | 5.86 | 4.91 | .13 |
| Participant Frustration Communicating | 5.07 | 1.91 | .22** |
| Participant Comfort Communicating | 6.00 | 2.00 | .26** |
| Resident Communication Factors | -1.10 | 0.71 | -.14 |

*Note*. For type of facility, comparison groups (1) are independent/assisted living and nursing home/skilled nursing, and reference group (0) is memory care unit.

**p* < .05. ***p* <.01. ****p* < .001.

**Supplementary Table 2.** *Multiple Regression Model: Social Visitation*

| Predictor | *B* | *SE* | ß |
| --- | --- | --- | --- |
| Years Living in Facility | -0.09 | 0.20 | -.03 |
| Minutes Traveled to LTC Facility | -0.01 | 0.00 | -.18* |
| Type of Facility: Independent/Assisted Living | 2.27 | 1.79 | .14 |
| Nursing Home/Skilled Nursing | 3.19 | 1.73 | .19 |
| Participant Frustration Communicating | 0.66 | 0.68 | .08 |
| Participant Comfort Communicating | 2.92 | 0.70 | .35*** |
| Resident Communication Factors | 0.18 | 0.25 | .06 |

*Note*. For type of facility, comparison groups (1) are independent/assisted living and nursing home/skilled nursing, and reference group (0) is memory care unit.

**p* < .05. ***p* <.01. ****p* < .001.

# **Survey Study**

## **Screener Question**

Do you know someone well, such as a close relative, friend, or loved one who currently resides in a long-term care (LTC) facility (e.g., retirement home, nursing home, assisted living facility)?

1. Yes
2. No
   1. If No: Thank you for your interest in completing this survey. You are not eligible to complete the rest of this survey at this time.

**Demographic Questionnaire**

The following questions ask for your demographic information:

1. What is your year of birth?
   1. *(drop-down menu)*
2. What is your gender?
   1. Male
   2. Female
   3. Other gender (please specify)
      1. *(text box)*
   4. Prefer not to say
3. Do you think of yourself as…?
   1. Lesbian or gay
   2. Straight
   3. Bisexual or pansexual
   4. Not listed above (please specify)

*(text box)*

- 1. Not sure

1. Which of the following best describes your race or origin? Please select all that apply.
   1. American Indian or Alaskan Native
   2. Asian
   3. Black or African American
   4. Hispanic, Latino or Spanish origin
   5. Middle Eastern, Arab, or North African
   6. Native Hawaiian or other Pacific Islander
   7. White
   8. Race and ethnicity unknown
   9. Race or ethnicity not listed here
   10. Prefer not to answer
2. What is your current relationship status?
   1. Single
   2. Long term relationship
   3. Married
   4. Widowed
   5. Separated
   6. Divorced
3. What is your employment status?
   1. Full-time
   2. Part-time
   3. Unemployed
   4. Retired
   5. Full-time Student
4. What is your household income range?
   1. Less than $25,000
   2. $25,000-$49,999
   3. $50,000-$74,999
   4. $75,000-$99,999
   5. $100,000-$149,999
   6. $150,000 or more
   7. Prefer not to say

**Family Involvement Questionnaire** (FIQ-LTC; Fast et al., 2019)

Please answer the following questions keeping in mind your closest family member, friend, or loved one who currently resides in a LTC facility.

The following questions ask about your involvement in your loved one's LTC facility. For each item please check the box that best indicates how often you engage in the activity in question.

Scale: Never (1), Rarely (2), Sometimes (3), Often (4)

|  | **Item** | **Subscale** |
| --- | --- | --- |
| 1. | I attend care conferences with staff to discuss my loved one’s general happiness and well-being. | (CC) |
| 2. | I contact my loved one’s facility if I have any questions. | (CC) |
| 3. | I talk with facility staff regarding my loved one’s eating habits. | (CC) |
| 4. | I inquired whether my loved one is engaging in community activities. | (CC) |
| 5. | I suggest possible activities to staff. | (CC) |
| 6. | I attend activities offered by my loved one’s facility. | **(S)** |
| 7. | I talk to facility staff about community and facility rules. | (CC) |
| 8. | I make sure my loved one has access to transportation. | (CC) |
| 9. | I ensure that my loved one has access to what they need for daily living. | (CC) |
| 10. | I speak with facility staff if I am concerned with something my loved one has said. | (CC) |
| 11. | I talk to facility staff to ensure my loved one has access to stimulating activities. | (CC) |
| 12. | I ensure that my loved one is satisfied with their level of privacy. | (CC) |
| 13. | I volunteer at my loved one’s facility. | **(S)** |
| 14. | I interact with my loved one during the holidays. | **(S)** |
| 15. | I talk to facility staff about my loved one’s engagement in their community. | (CC) |
| 16. | I bring or send my loved one gifts. | **(S)** |
| 17. | I talk to facility staff about my loved one’s friends and social life. | (CC) |
| 18. | I visit my loved one in their long-term care facility. | **(S)** |
| 19. | I talk to facility staff about problems my loved one may be experiencing. | (CC) |
| 20. | I talk to my loved one about how their day was. | **(S)** |
| 21. | I encourage my loved one to engage in social activities. | **(S)** |
| 22. | I talk to other people who have loved ones in the same facility. | (CC) |
| 23. | I make sure my loved one has the means to easily move around their facility. | (CC) |
| 24. | I give input into my loved one's care plan. | (CC) |
| 25. | I communicate with my loved one over the internet. | **(S)** |
| 26. | I try to help my loved one transition into living in a long-term care facility. | **(S)** |
| 27. | I feel that facility staff encourage my loved one to interact with residents. | (CC) |
| 28. | I come and have meals with my loved one. | **(S)** |
| 29. | I help my loved one with tasks they may be struggling with. | (CC) |
| 30. | I talk to my loved one about their interests. | **(S)** |
| 31. | I listen to my loved one’s concerns regarding their facility. | **(S)** |
| 32. | I communicate with my loved one through letters. | **(S)** |
| 33. | I keep up to date on my loved one’s health status. | (CC) |
| 34. | I contact facility staff by phone or email. | (CC) |
| 35. | I talk about how my loved one is doing with facility staff or their family. | (CC) |
| 36. | I assist my loved one in managing their finances. | (CC) |
| 37. | I provide my loved one with picture frames or wall decorations. | **(S)** |
| 38. | If my loved one mentions one of their personal items is missing, I speak to facility staff about their concern. | (CC) |
| 39. | I participate in family council. | (CC) |
| 40. | I talk with facility staff about problems they feel my loved one may be experiencing. | (CC) |

(CC): Care Coordination Involvement Subscale Items: 1-5, 7-12, 15, 17, 19, 22-24, 27, 29, 33-36, 38-40. Scores range from 26 to 104.

**(S)**: Social Visitation Subscale Items: 6, 13, 14, 16, 18, 20, 21, 25, 26, 28, 30-32, 37.

Scores range from 14 to 56.

*Note: Subscales were created by the authors for the purpose of this study.*

Fast, C. T., Houlihan, D., & Buchanan, J. A. (2019). Developing the family involvement questionnaire-long-term care: a measure of familial involvement in the lives of residents at long-term care facilities. *The Gerontologist*, *59*(2), e52-e65.

**LTC Facility Characteristics Questionnaire**

Below is a list of questions about the person you know well, such as a family member, friend, or loved one, who is currently residing in a long-term care (LTC) facility.

**If you know more than one person currently residing in a LTC facility, please respond regarding the individual you know the best or are closest with.**

1. What type of long-term care facility does your family member, friend, or loved one reside in?
   1. Independent Living Home / Apartment
      1. Private residence equipped with home amenities such as housekeeping and transportation, but not equipped with medical services.
   2. Assisted Living Facility
      1. For older adults who need help with their daily routines, but who do not need 24-hour care.
   3. Nursing Home / Skilled Nursing
      1. Staffed 24-hours with skilled nurses to assist with bathing, dressing, eating, and medication management.
      2. This is what comes to mind for most people when they think of LTC Facilities.
   4. Memory Care Unit
      1. Specialized care unit for people living with advanced dementia.
   5. I don’t know
   6. Other/Comments:_________
2. How long has your family member, friend, or loved one been living in the LTC facility? If unsure, please give your best guess.
   1. Sliding scale number of months.
      1. Number of **months** your loved one has lived in the LTC facility.
         - *Note:*24 months = 2 years; 36 months = 3 years, 48 months = 4 years, 60 months = 5 years
   2. If 5+ years is selected
      1. How many total years has your family member, friend, or loved one lived in the LTC facility? If unsure, please give your best guess.
         - (text-based response)
3. How many minutes does it take for you to travel to your family member, friend, or loved one residing in a LTC facility?
   1. (0 - 500)
      1. “*Note*: 4 hours = 240 minutes; 5 hours = 300 minutes; 6 hours = 360 minutes; 7 hours = 420 minutes; 8 hours = 480 minutes)
      2. More than 8 hours.

**Resident Communication Factors Questionnaire**

1. Is your family member, friend, or loved one currently able to communicate verbally?
   1. Never
   2. Rarely
   3. Sometimes
   4. Very often
   5. Always
   6. I don’t know
2. Does your family member, friend, or loved one currently recognize you when you are together?
   1. Never
   2. Rarely
   3. Sometimes
   4. Very often
   5. Always
   6. I don’t know
3. How often is your family member, friend, or loved one currently alert and aware of your interactions together?
   1. Never
   2. Rarely
   3. Sometimes
   4. Very often
   5. Always
   6. I don’t know

**Visitor Communication Experience Questionnaire**

1. How comfortable are you communicating and interacting with your family member, friend, or loved one?
   1. Not at all comfortable
   2. Slightly comfortable
   3. Somewhat comfortable
   4. Very comfortable
   5. Extremely comfortable

2. How frustrated do you become when interacting with your family member, friend, or loved one?

- 1. Not at all frustrated
  2. Slightly frustrated
  3. Somewhat frustrated
  4. Very frustrated
  5. Extremely frustrated
